# Proprioception is sufficient to coordinate simple bimanual lifts of unanticipated asymmetrically loaded objects

**DOI:** 10.1007/s00221-026-07377-9

**Published:** 2026-08-24

**Authors:** Warren G. Darling, Anna C. Snyder, Lavena Mikhail

**Affiliations:** Department of Health, Sport and Human Physiology, Motor Control Laboratory, University of Iowa, 225 S. Grand Avenue, Iowa City, IA 52242 USA

**Keywords:** Proprioception, Vision, Bimanual, Movement

## Abstract

The relative importance of proprioception and vision in control of bimanual lifting of a box with varied weight and weight distribution was studied to test whether availability of vision improved the lifting motion in terms of box orientation and temporal synchrony of lifting the two sides of the box. Twelve right-handed young adults performed a random sequence of bimanual lifts of a box with varied weight and weight distribution. When lifting a symmetrically loaded box there was excellent temporal synchrony of the motions of the left and right side and minimal deviation from a level box orientation. When lifting an asymmetrically loaded box there was delayed onset of lifting the heavier side of the box but temporal synchrony of right and left side motions subsequently improved such that left and right sides ended motion nearly synchronously. There was also greater deviation from a level box orientation during the lift that was corrected to nearly level at lift termination. Adaptations to changes in load symmetry from heavier load on one side to the other side occurred over 2–3 lifts to produce nearly synchronous lifts of the left and right sides with little deviation from a level box orientation. Allowing vision did not improve maintenance of box orientation or temporal synchronization of left and right side lifting motions. These results show that non-visual information is sufficient to control lifting a box to a level orientation when there is no prior knowledge of box weight or its distribution within the box.

## Introduction

The relative importance of different types of sensory information in lifting a single object with both hands, a common everyday task, has received remarkably little scientific investigation. In some tasks, the object weight and its distribution are known in advance or estimated based on visual information and past experience with the object, thereby permitting anticipatory control of the applied lifting forces by each hand (e.g., Bracewell et al. 2003). In contrast, when such information is not available until the lift begins, subjects must adjust applied lifting forces with one or both hands based on sensory information about the motion of the hands and object as it is lifted. Both proprioception and vision can provide sensory information for use in controlling such lifts but it is not known whether proprioception alone provides sufficiently precise awareness of hand/object motion to produce a lift with minimal tilt of the object, which is the usual goal. Of course, proprioception is always available in neurologically intact individuals but it is not known whether lifts are performed better (i.e., quicker and with less tilt of the object) when vision is allowed due to the additional sensory information about hand motion and object tilt. Here we studied the kinematics of lifts of a relatively large object with varied weight and weight distribution to mimic everyday tasks such as lifting a box when the lifter cannot see its contents.

Previously it was assumed that proprioceptive sensations were too noisy to provide accurate estimates of limb orientation and motion for precise control of movements (e.g., Wolpert et al. 1995; Wolpert and Ghahramani 2000). However, a number of more recent studies demonstrated that proprioceptive information alone can be used to guide accurate hand movements to stationary and moving internal (body) targets (Capaday et al. 2013; Darling et al. 2018, 2024; Coffman et al. 2021; Yoss et al. 2022; Darling and Yem 2023; Darling and Zuck 2024). Indeed, it was reported in those studies that the level of precision of proprioceptive guidance to internal targets was close to or sometimes better than when vision was allowed during such movements. Whether proprioceptive information alone can be used to appropriately control bimanual lifts of relatively large objects of unknown weight and weight distribution has not been studied previously. Indeed, most previous studies of bimanual lifting have investigated simultaneous bimanual lifts of two similar separate small objects to study bimanual interactions between the applied grip and load (lift) forces to each object by the index and thumb tips of both hands (e.g., Serrien and Wiesendanger 2001; Steenbergen et al. 2008; Dimitriou and Buckingham 2018). Unexpected lower weight of one of the objects resulted in a higher than needed grip force applied to the lighter object, reflecting the expectation of a larger weight. In contrast, unexpected increases in weight of one of the objects did not affect applied grip force, presumably because the grip force continued to increase along with load force to produce the higher load force needed to lift the heavier object (Serrien and Wiesendanger 2001). Bimanual lifting of a single small object by the right and left index tips has also been studied previously, but only with unexpected variations in the surface friction of each side (Edin et al. 1992; Burstedt et al. 1997). Normal/tangential (grip/lift) force ratios were scaled according to the friction experienced by each index-tip such that higher tangential (lift) force was applied to the higher friction (less slippery) surface and the object tilted slightly away from that side. Notably, digital nerve block demonstrated that cutaneous receptors of the index tips were necessary to appropriately adjust the normal/tangential force ratios according to friction.

When applying forces with both hands to a single object, an unexpected lower weight distributed to one side results in an earlier onset and faster lift of that side and tilt of the object which can be counteracted by increasing the lifting force applied to the other (heavier) side and/or by reducing the lifting force applied to the lighter side. In such tasks, visual and/or proprioceptive/cutaneous information about object tilt and position/ motion of the two hands/sides of the object may be used to initiate compensatory application of lifting forces of one or both hands to prevent excessive object tilt during and at the end of the lift. Cutaneous information about the applied lifting force to each side may also contribute to such compensatory actions. In an initial study of bimanual lifting of a single object with asymmetric weight distribution, it was shown that subjects varied hand placement and applied forces with each hand based on past experience (practice lifting trials were provided) to compensate for asymmetric weight distribution and minimize object roll (tilt) during the lift (Lee-Miller et al. 2019). All trials were conducted with vision allowed, providing subjects with visual information about the amount of object tilt, speed of hand motions and location of hand placement. Of course, proprioceptive and cutaneous sensations were also available, thus the relative importance of these different sources of sensory information in controlling the lift was not investigated. It was shown that subjects placed the corresponding hand higher on the side with higher load distribution and exerted larger lift forces on that side to prevent tilt of the box. Although not quantified, it appears in Fig. 2 of Lee-Miller et al. (2019) that lift (load) and grip forces were initiated nearly synchronously with the two hands and the peak lift and grip force rates were also synchronous. This would be consistent with many previous reports of temporal synchrony and spatial assimilation in rhythmic and discrete bimanual reaches to separate visual targets and drawing movements (e.g., Kelso et al. 1979a; Kelso et al. 1979b; Franz et al. 1991; Franz 1997; Franz et al. 2001; Marteniuk et al., 1984).

Previous studies of the role of proprioception in bimanual reaching tasks have shown that proprioception contributes to temporal synchronization and direction of movements of the two hands. For example, mechanical perturbations applied to one upper limb during bimanual control of a single cursor on a display result in reflexive EMG responses in both upper limbs that contribute to achieving the movement goal (Mutha and Sainburg 2009). The onset of these bimanual EMG responses at about 50 ms after the perturbation suggest an origin based on stimulation of proprioceptive or cutaneous receptors. Visual responses to perturbations of target location and to visually presented hand location have much longer latencies in both deafferented and neurologically intact individuals (e.g., Jayasinghe et al. 2024).

The primary purpose of the current investigation was to determine whether availability of vision improves control of comfortable speed bimanual lifts of a single object with symmetrical and asymmetrical weight distributions under expected and unexpected conditions. We hypothesized that when weight distribution was predictable from previous experience that lifting of the two sides of the object would be nearly synchronous and object tilt would be minimal whether vision was available or not. When weight and its distribution was not predictable, we expected: (1) nearly synchronous lift of the two sides with minimal tilt when weight distribution was symmetric whether vision was available or not (i.e., a plausible bimanual coordination plan would be similar applied lifting forces to each side of the object) and (2) asynchronous lift of the two sides (later onset to lift the heavier side) but synchronous lift termination with larger object tilt during the lift and at termination than in symmetrical load conditions whether vision was available or not. Time delay to initiate lift of the heavier side was expected to be similar in no vision and vision conditions due to the shorter time to process proprioceptive than visual information (e.g., Bracewell et al. 2003). However, when vision was available we hypothesized that object tilt during and at the end of the lift would be lower than when vision is blocked and only proprioception/cutaneous information was available because movement durations were sufficient for vision to contribute to leveling the box during the lift. We also examined the effects of transitions from an asymmetric right-sided load to consecutive lifts of an asymmetric left-sided load and vice versa on lifting performance to assess adaptation to changes in load symmetry. We hypothesized that rapid adaptation to produce more synchronous lifts would occur but that adaptation to minimize object tilt would be better when vision was allowed.

## Methods

### Subjects

Twelve right-handed healthy young adults (18–35 years, 6 males) served as subjects in these experiments. All subjects provided verbal consent after reviewing informed consent documents approved by the University of Iowa Institutional Review Board.

### Apparatus

**Fig. 1:**
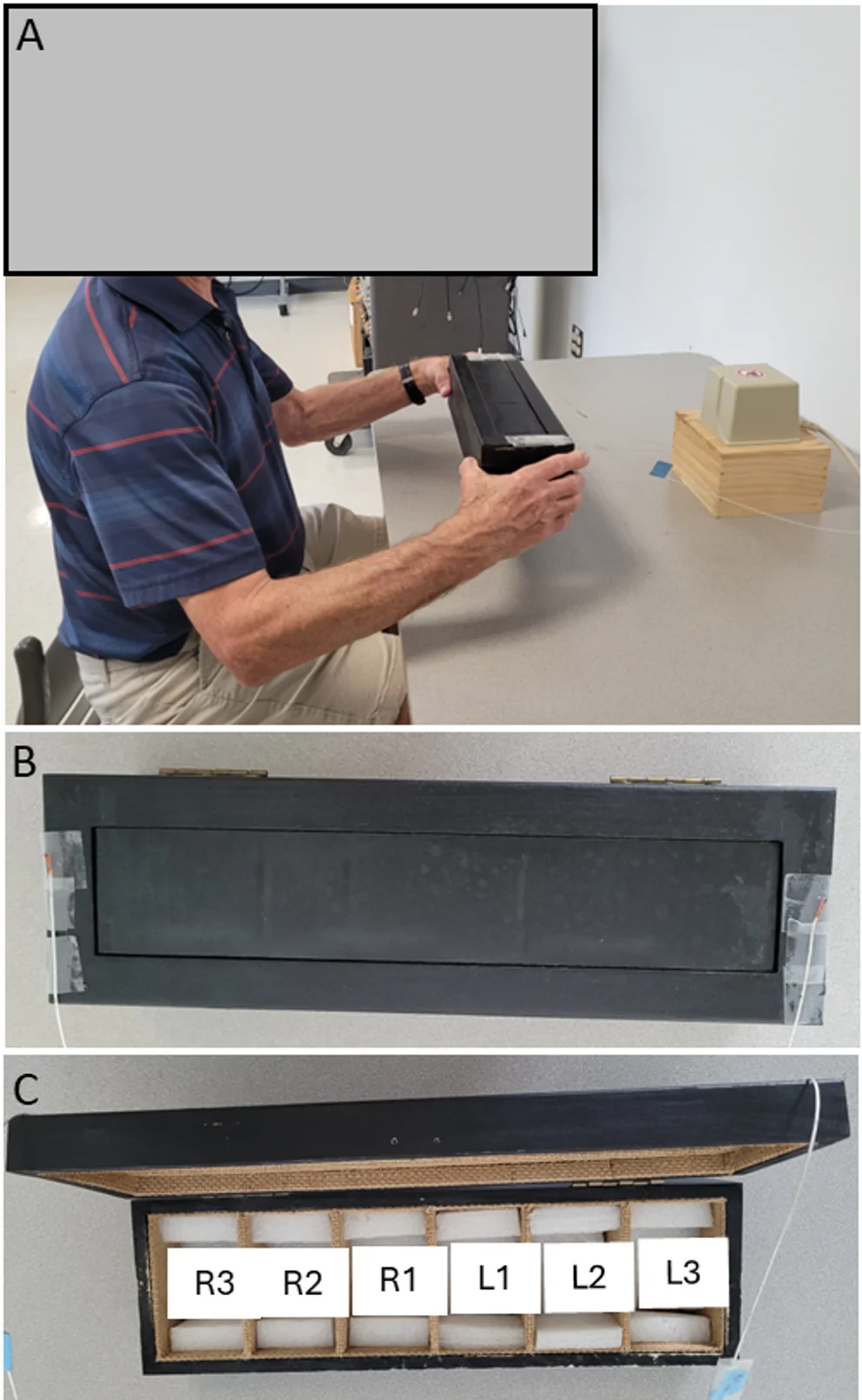
Experimental setup (**A**) and modified watch box (**B**) with six compartments (**C**) labeled L3, L2, L1, R1, R2, R3. Model 130 Trakstar sensors were taped to the right and left sides of the lid of the box. Box opened away from participant and was closed whenever the subject had vision available

Three-dimensional location/motion of a modified watch box (36 cm × 9 cm × 9 cm) weighing 700 g (6.87 N) (Fig. 1B,C) were measured using an electromagnetic system (Northern Digital 3D Guidance Trakstar). Small cylindrical (1.5 × 5.7 mm) 6D magnetic position/orientation sensors (model 130, position accuracy 1.4 mm RMS) were attached to each side of the modified watch box (Fig. 1) to measure vertical motion of each side at 240 samples/s (sufficient to capture temporal information during hand movements) with a custom MATLAB program. The watch box permitted placement of 0 g, 100 g (0.981 N), 200 g (1.962 N) and 400 g (3.924) masses (weights) in different locations such that the weights were distributed symmetrically or asymmetrically. Additional weight for each lift could vary between 1.962 and 7.848 N such that total weight lifted varied between 8.832 and 10.794 N. Each box contained six compartments labeled L3, L2, L1, R1, R2, R3 from left to right that were cushioned to reduce sound when weights were placed into the box (Fig. 1C). White noise was played throughout the experiment to mask placement of weights.

Weights were placed into the box by one experimenter according to fixed and pseudo-random (hereafter referred to as random) sequences in different conditions. A second experimenter controlled data collection and, when the first experimenter said “Ready” to indicate weights were placed, would initiate data collection and say “Go” to tell the subjects to lift the box. An initial fixed sequence with symmetric weight distribution allowed subjects to experience/practice lifting the box with different total weights (i.e., added 200 g, 400 g, 800 g) before a sequence with random variations in weight and weight distribution. In the initial 9 trial sequence with symmetric weight distribution there were 3 consecutive lifts with each of the different weights for a total of 9 lifts (i.e., 3 with 200 g added load, 3 with 400 g added load and 3 with 800 g added load). This sequence was labelled “training” in figures in the Results section. This was followed by a random sequence of 54 lifts in which variations in total weight and weight distribution occurred (i.e., lifts with symmetrical loads of 200/400/800 g added load, lifts with 200/400/800 g added load on the left side, lifts with 200/400/800 g added load on the right side). There were never two consecutive lifts with the same weight or weight distribution, thus subjects could not anticipate the total weight or weight distribution for each lift. The final lift of the random sequence had 400 g added load distributed to the right side of the box. This random sequence was then followed by four fixed sequences: (1) five lifts with 400 g distributed to the left side of the box, (2) five lifts with 400 g distributed to the right side of the box, (3) 5 lifts with 800 g distributed to the left side of the box and (4) 5 lifts with 800 g distributed to the right side of the box. All the lift sequences (i.e., 9 trials of symmetric lifts, 54 lifts with random weight and weight distribution, four fixed load asymmetry lifts)were first completed with either vision permitted during the lift (six subjects, one experimenter verified eyes were closed during placement of weights) or with subjects blindfolded throughout (six subjects). Following completion of this first set of 83 lifts the entire group of sequences was repeated but subjects who had vision initially were blindfolded for the second set and those who were blindfolded initially had vision allowed during the lifts (one experimenter verified eyes were closed during placement of weights) for the second set.

### Experimental design and protocol

Subjects sat in a chair at a desk with the watch box located directly in front of them (Fig. 1A). After weights were placed the subject would place their hands on each side of the box and Experimenter 1 would say “Ready” to notify experimenter 2 that the lift could begin. Experimenter 2 would then initiate data collection and say “Go” for the subject to begin the lift. Subjects placed their hands next to the sides of the box and were instructed to grasp the sides of the box with the thumb on the edge closer to the subject and fingers on the edge further from the subject (Fig. 1) and to lift the box smoothly in a single movement to a level orientation about 6–12 inches (15–30 cm) above the desk. They were also instructed to not correct the box orientation after the end of the first movement. The independent variables in this experiment were order (vision first or no-vision first), availability of vision, and experimental condition (training-symmetric, random-symmetric/asymmetric). Fixed asymmetric lifts were analyzed separately. There were two different random-asymmetric conditions (right load distribution, left load distribution) with varying degrees of load asymmetry (i.e., 200 g, 400 g or 800 g distributed to the right or left) along with random symmetrical load lifts (200 g, 400 g, 800 g) for a total of 54 lifts (i.e., 6 lifts of each type – 18 lifts with symmetrical loads randomly interspersed with 6 lifts of each of the 6 types of asymmetrical load). This series of 54 lifts was followed by four different fixed-asymmetric conditions with 5 consecutive lifts with the same load distribution that were preceded by a different asymmetrical load lift as follows: (1) 400 g to left followed by 5 lifts of 400 g to right (L400-R400), (2) 400 g to right followed by 5 lifts of 400 g to left (R400-L400, (3) 400 g to left followed by 5 lifts of 800 g to right (L400-R800), (4) 800 g to right followed by 5 lifts of 800 g to left (R800-L800). Subjects were not informed that there would be five consecutive lifts with the same load and asymmetry or when the loading was changed.

Dependent variables were differences between left and right sides of the box for: lift onset time, time of peak upward velocity, time of lift termination. The range of box tilt motion during the lift and box tilt at lift termination were also measured to assess how well subjects maintained the box in a level orientation during the lift and accuracy of the final orientation of the initial lift. Corrections to final box orientation after termination were not included in the analysis.

### Data analysis

**Fig. 2:**
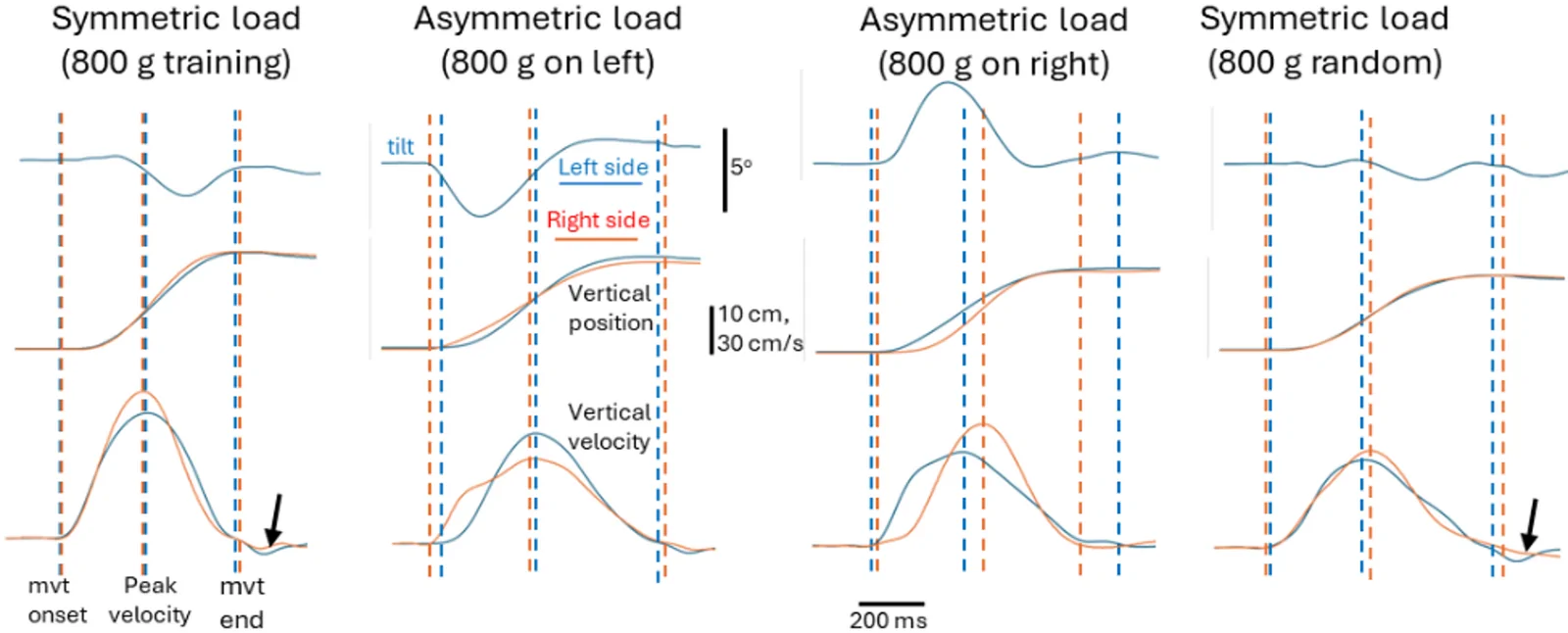
Examples of vertical position and velocity profiles of the right and left sides of the box and tilt angle of the box during lifts under the different load conditions in one subject while blindfolded. Asymmetrical load lifts were performed in random conditions only. Right side vertical position and velocity are shown in orange. Left side vertical position and velocity and box tilt shown in blue. Movement onsets, times of peak velocity and movement terminations are shown as dotted lines. The arrows on the vertical velocity profiles show secondary movements that were not included in the analysis of tilt accuracy at the end of the lift

Kinematic motion data of the left and right sides of the box were analyzed in datapac 2k2 (Run Technologies, Mission Viejo, CA, USA). 3D position data were digitally filtered (lowpass filtered, 15 Hz rolloff frequency, no phase lag). Onset and termination of the lift of the left and right sides of the box were determined using a vertical velocity threshold of 2.3 cm/s and verified by visual inspection (e.g., Fig. 2). Lift termination was adjusted visually to the end of the movement if a secondary upward movement began shortly before vertical velocity decreased below 2.3 cm/s (e.g., Fig. 2 – note secondary movements shown by arrows after the first movement was terminated). Corrective movements sometimes occurred after termination, but were not included in the analysis. Peak upward velocities of the left and right sides of the box were measured as the maximum upward velocity between lift onset and termination. Range of box tilt motion during the lift was measured from the maximum and minimum difference in vertical position of the left and right sides of the box between lift onset and termination. Tilt angle of the box was computed from the difference in vertical position and the distance between the sensors on the left and right sides of the box (Eq. 1) at these times and at termination of the lift (i.e., later time of end of upward motion of the left and right sides of the box).

1 $$ {\mathrm{Tiltang}} = {\text{arc sin}}\left( {\left( {{\mathrm{Z}}_{{\mathrm{L}}} {-}{\mathrm{Z}}_{{\mathrm{R}}} } \right)/{\mathrm{D}}} \right) $$

Where Tiltang is tilt angle, Z_L_ is vertical position of the left side of the box, Z_R_ is vertical position of the right side of the box and D is distance between the left and right side sensors.

Trials in consecutive symmetrical loading conditions during training were analyzed to compute the mean and variability (SD) of each dependent variable for comparison with symmetrical loading trials occurring in the random loading conditions. All trials in random loading conditions were sorted into symmetrical load, left asymmetrical load (more weight on left side) and right asymmetrical load (more weight on right side). Mean and variability (S.D.) of each dependent variable were computed for each subject to assess temporal synchronization of lifting the left and right sides of the box under initial symmetric training conditions and under random conditions when blindfolded and with vision allowed.

Trials in the fixed asymmetrical load conditions were analyzed to assess subjects’ abilities to deal with transitions of load asymmetry on the first attempt and second attempt with the new loading condition. The average of the last 3 trials of each fixed asymmetric condition provided a measure of subjects’ abilities to temporally synchronize right and left side lift onset times, time of peak velocity and time of movement termination after two trials of experience with the asymmetrically loaded box. Specifically, we were interested in determining whether performance improved from the first to the second lift and whether there was evidence of further improvement after the 2nd lift.

### Statistical analyses

Means and standard deviations of each dependent variable for each subject were compiled for each condition (i.e., symmetrical load in fixed and random orders, left asymmetrically loaded and right asymmetrically loaded in random order). Each dependent variable was submitted to a 3-way (order, vision availability, type of sequence) repeated measures analysis of variance (ANOVA) to test for synchronicity of temporal variables of the left and right side lifts, differences in peak upward velocity of the left and right sides of the box and of spatial variables related to accuracy of lift (i.e., maintaining the box in a level orientation). Huynh–Feldt corrections for sphericity violations were applied to p-values when necessary and corrected p-values are reported in the Results section). Differences in times of lift onset, peak upward velocity and lift termination were analyzed along with differences in peak upward velocity of the left and right sides of the box, tilt range of motion (ROM) during the lift and box tilt at lift termination. Variability (S.D.) of each dependent variable was also analyzed using 3-way repeated measures ANOVA to test whether variability was lower in symmetric versus asymmetrically loaded conditions and fixed versus random sequences for the different load conditions. If the order main effects in these three-way repeated measures ANOVAs were not statistically significant (p > 0.05) a two-way (vision availability, type of sequence) repeated measures ANOVA was applied. However, there were no order main effects (p > 0.289) for any of the timing dependent variables or for accuracy of the lifts (final tilt angle), thus only two-way repeated measures ANOVAs are reported for those results. Because the 3 temporal variables and their variability may be correlated, we performed Bonferroni corrections to maintain a family-wise error rate with p < 0.05. Specifically, among the 6 temporal variables p-values less than 0.05/6 or 0.083 were required for statistical significance. Final tilt and tilt ROM may also be correlated, thus among the 4 tilt variables p-values less than 0.05/4 or 0.0125 were required for significance. We also computed effect sizes (Cohen’s ƒ) for the main effects and interactions of interest.

The fixed sequence asymmetrical loads following a transition from right/left side asymmetrical load to left/right side asymmetrical load were analyzed using the same dependent variables for the 1st trial, 2nd trial and average of trials 3–5. The purpose of this analysis was to examine temporal synchronicity at movement onset, time of peak velocity and movement termination as well as tilt angle (ROM and at movement termination) of the 2nd trial and trials 3–5 in comparison to the first trial. These variables were initially entered into four-way (order of performing first with or without vision, vision availability, type of transition, trials) repeated measures ANOVAs to assess statistical changes in performance in dealing with transitions from left to right load asymmetry and vice-versa. In all but one dependent variable (tilt range of motion during the lift) – F_1,10_ = 9.59, p = 0.011), there was no effect of order of performing with vision first versus blindfolded first as a main or interaction effect (p > 0.08). Thus, results of the three-way (transition type x vision x trial) repeated measures ANOVA are reported for most dependent variables. Bonferroni corrections were also applied for the vision main effects and interactions as described in the previous paragraph.

## Results

### Kinematic characteristics of the lifts

**Fig. 3:**
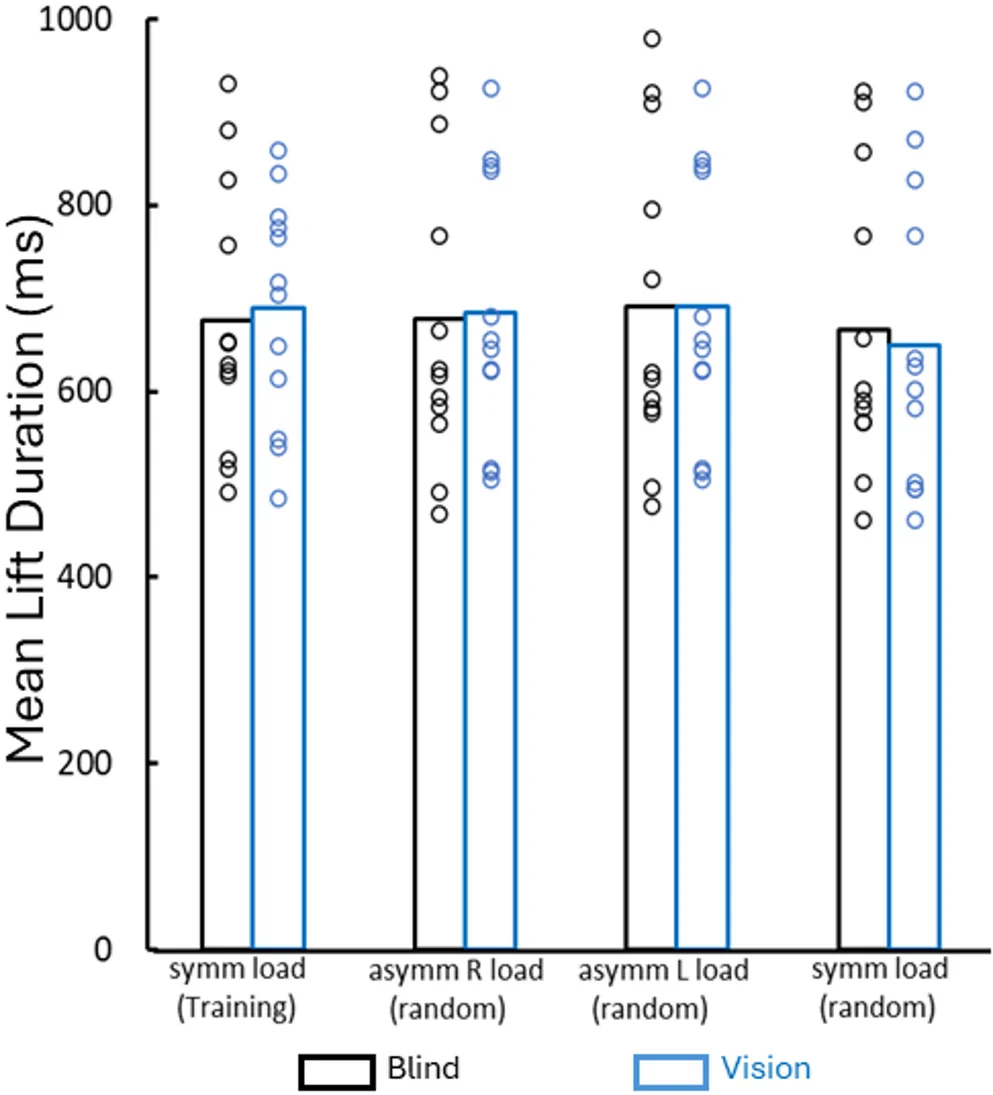
Mean durations of lifts of symmetrical loads performed in the training and random conditions and of lifts of asymmetrical loads in the random conditions. Each bar is the mean of 12 subjects’ movement durations with vision allowed (blue) or while blindfolded (black). The plotted symbols are individual subjects’ mean movement durations

Lifting movements were generally smooth and, for symmetrical loads, were accomplished with bell-shaped single-peaked velocity profiles that were approximately temporally synchronous with similar peak velocities in both training (all symmetrical loads) and under random conditions (e.g., Fig. 2 – symmetrical loads). In contrast, when lifting asymmetrical loads in random conditions the lift of the lighter side was less smooth, sometimes with a double peaked velocity profile, while the heavier side velocity profile was not synchronized to the lighter side (e.g., Fig. 2 – asymmetrical loads. Total movement durations (i.e., from the time of first upward velocity of the right or left side to later time of movement termination by the left or right side) averaged about 700 ms for movements made with and without vision available in the training and random loading conditions (Fig. 3).

### Temporal synchronization of right-left lift onsets

**Fig. 4:**
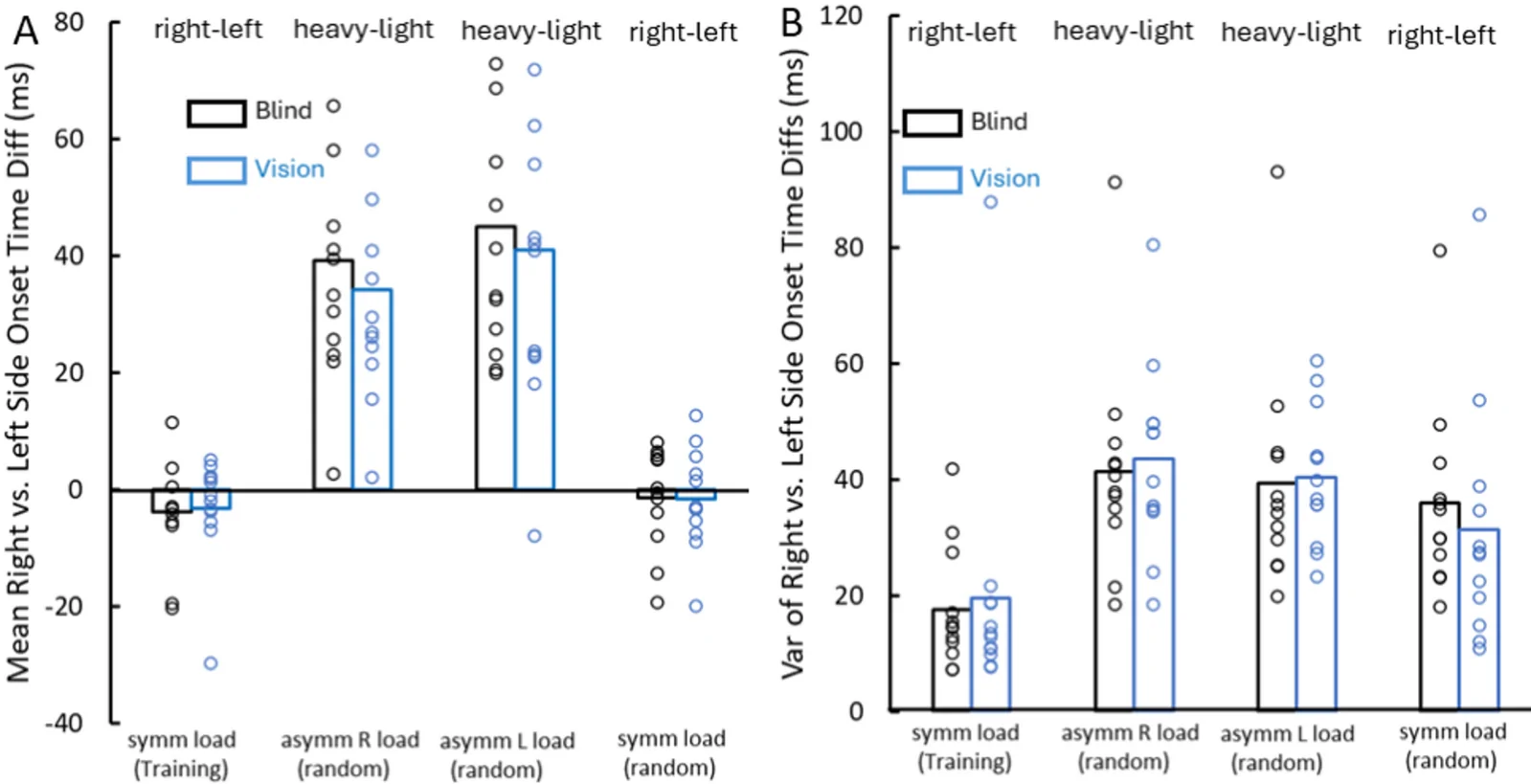
Mean right-left side lift onset time differences (**A**) and variability (SD) of those differences (**B**) in each lifting condition. Each bar in A is the mean onset time difference (right-left for symmetrical loads, heavy side – light side for asymmetrical loads) for 12 subjects in vision (blue) and blindfolded (black) conditions. Each bar in B is the mean of the S.D.s of onset time differences (right-left for symmetrical loads, heavy side – light side for asymmetrical loads) for 12 subjects in vision (blue) and blindfolded (black) conditions. Individual subject means and S.D.s are shown by the symbols. The different lifting conditions are shown on the abscissa. Individual subject data are shown by the symbols plotted with each bar

Lifts of the left and right sides of the box were nearly synchronous when the box was symmetrically loaded in both fixed (training) and random conditions but were asynchronous when loads were asymmetric. This is clearly shown in the single trial examples from one subject (Fig. 2 – compare the lifts of symmetrical loads to those of asymmetrical loads). Across all subjects, differences between left and right side lift onset times averaged less than 5 ms when lifting symmetrical loads in both training and random conditions and with and without vision (Fig. 4A). In contrast, during asymmetric conditions the onset time for lifting the heavy side was delayed by, on average, more than 30 ms in both vision and no-vision conditions (Fig. 4A)... Statistical analysis showed there was no effect of vision availability on temporal synchrony of left/right onset times (Fig. 4A, F_1,11_ = 0.36, p = 0.559, ƒ = 0.182) but asymmetrical loads had longer onset time differences for the heavier side of the box (F_3,33_ = 26.26, p < 0.001, ƒ = 1.545, p < 0.001 on post-hoc tests comparing lifts of asymmetric and symmetrical loads). There was no interaction of vision availability and type of load (F_3,33_ = 0.78, p = 0.513, f = 0.266,).

As expected, variability of onset time differences averaged about 2X higher when loads and load symmetry were varied in random sequence lifts than in the training condition when all loads were symmetric (Fig. 4B). Statistical analysis showed that variability of right and left side onset time differences were similar in vision and blindfolded conditions (Fig. 4B, F_1,11_ = 0.23, p = 0.942, ƒ = 0.145) but were higher for lifts performed in random conditions than in training when only symmetrical loads were lifted (Fig. 4B, F3,33 = 26.61, p < 0.001, ƒ = 1.555; p < 0.007 for post-hoc comparisons of variability in training condition versus random symmetric and asymmetrical loading conditions). Under random conditions, variability of onset time differences was similar whether the heavier load was on the left or right side (Fig. 4B, p = 0.464). Variability of onset time differences was lower for lifting symmetrical loads than for lifting asymmetrical loads in the random condition (Fig. 4B, p = 0.007) but not for lifting left-sided asymmetrical loads (Fig. 4B, p = 0.191). There was no interaction of vision availability and type of loading (F_3,33_ = 0.36, p = 0.784, ƒ = 0.180).

## Temporal Synchronization of right-left peak velocities

**Fig. 5:**
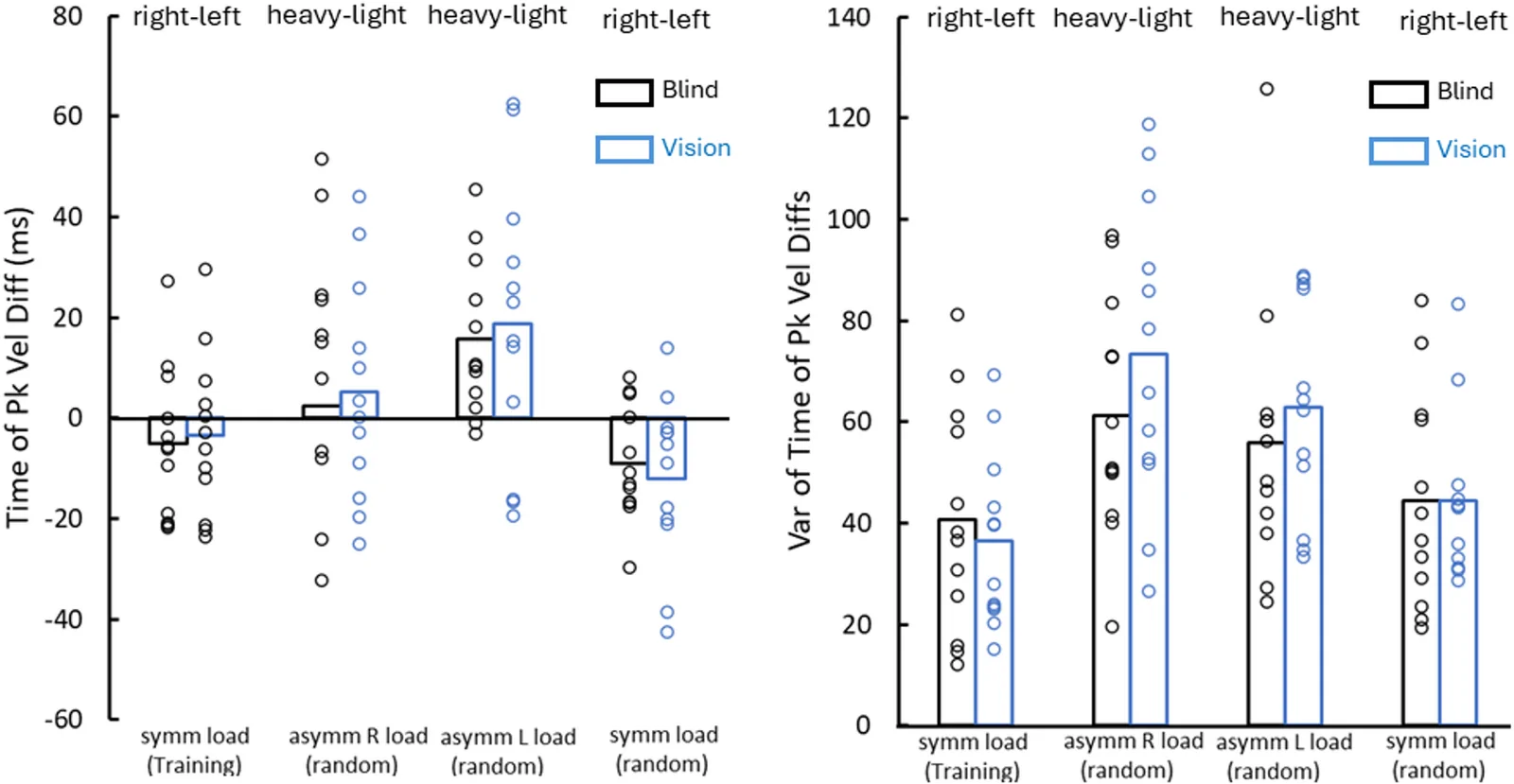
Mean right-left side lift time of peak velocity differences (**A**) and variability (SD) of those differences (**B**) in each condition. Each bar in A is the mean time of peak velocity difference (right-left for symmetrical loads, heavy side – light side for asymmetrical loads) for 12 subjects in vision (blue) and blindfolded (black) conditions. Each bar in B is the mean of the S.D.s of time peak velocity differences (right-left for symmetrical loads, heavy side – light side for asymmetrical loads) for 12 subjects in vision (blue) and blindfolded (black) conditions. Individual subject data are shown by the symbols plotted with each bar

Times of peak velocity for lifting the right and left sides of the box were nearly synchronous for lifting symmetrical loads but usually differed for lifting the heavier side of asymmetrical loads (e.g., Fig. 2). Analysis of all subjects’ data showed that availability of vision did not affect right-left or heavy-light side differences in timing of peak velocity (Fig. 5A, F1,11 = 0.58, p = 0.462, ƒ = 0.230). However, peak velocity times were generally closer to synchronous (i.e., time difference of 0.0 ms) when lifting symmetrical loads and asymmetrical loads weighted to the right than when lifting asymmetrical loads weighted to the left (Fig. 5A, F3,33 = 10.93,p < 0.001, ƒ = 0.997, p < 0.05 for comparing symmetrical loads to asymmetric left load, p = 0.136 for comparing lifts of left and right asymmetrical loads). There was no interaction of vision and type of loading effects on right-left differences in time of peak velocities (F_3,33_ = 1.63, p = 0.202, ƒ = 0.384).

Variability of differences in timing of peak velocities averaged less than 50 ms for lifting symmetrical loads in training and random conditions and averaged about 60–70 ms when lifting asymmetrical loads in random conditions. Availability of vision did not affect variability of right-left differences in timing of peak velocity (Fig. 5B, F1,11 = 0.53,p = 0.480, ƒ = 0.220) but this variability was higher when lifting asymmetrical loads (Fig. 5B, F3,33 = 11.95,p < 0.001, ƒ = 1.04; p < 0.05 for post-hoc comparisons of lifting asymmetrical loads in random conditions versus lifting symmetrical loads in training and random conditions). Variability of right-left timing of peak velocity was similar for lifting symmetrical loads in training and random conditions (Fig. 5B, p = 0.694 on post-hoc comparison). There was no interaction of vision and type of loading effects on variability of right-left differences in timing of peak velocity (F_3,33_ = 0.94, p = 0.435. ƒ = 0.292).

### Temporal synchronization of right-left movement terminations

**Fig. 6:**
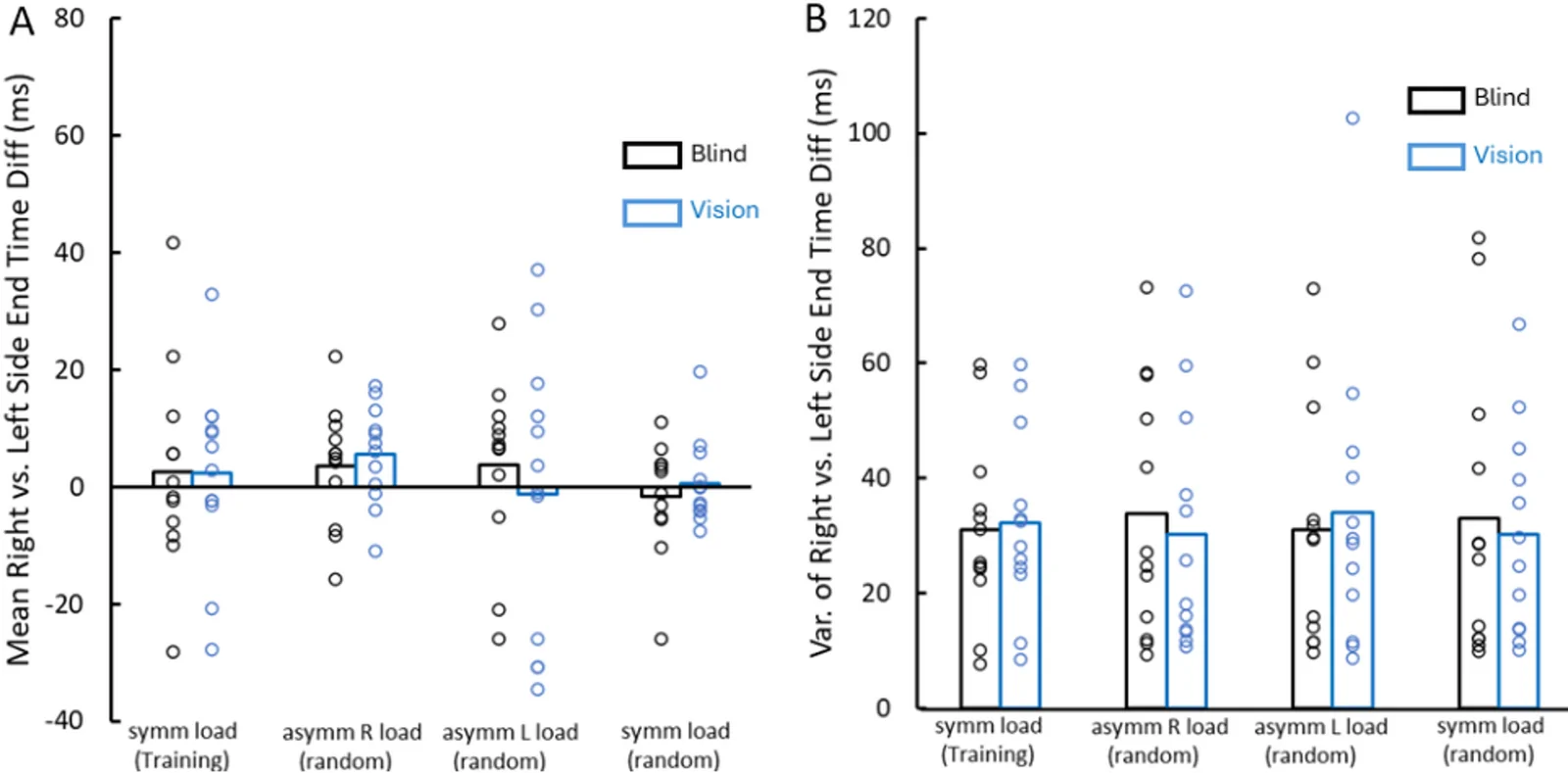
Mean left–right side lift movement end time differences (**A**) and variability (SD) of those differences (**B**) in each condition. Each bar in A is the mean end time difference (right-left for symmetrical loads, heavy side – light side for asymmetrical loads) for 12 subjects in vision (blue) and blindfolded (black) conditions. Each bar in B is the mean of the S.D.s of onset time differences (right-left for symmetrical loads, heavy side – light side for asymmetrical loads) for 12 subjects in vision (blue) and blindfolded (black) conditions. Individual subject data shown by the symbols plotted with each bar

Right and left side movement terminations averaged within 50 ms of each other when lifting symmetrical loads in training and both asymmetric and symmetrical loads in random conditions (Fig. 6A). Availability of vision (Fig. 6A, F1,11 = 0.02, p = 0.897, ƒ = 0.040) and type of loading (F_3,33_ = 0.41, p = 0.616, ƒ = 0.194) did not affect synchrony of movement termination, nor was there an interaction of these effects (F_3,33_ = 0.35,p = 0.789, ƒ = 0.178). Similar statistical results were found for variability of right-left differences in movement terminations (Fig. 6B, F1,11 = 0.038,p = 0.849, ƒ = 0.059 for Vision availability; F_3,33_ = 0.02, p = 0.974, ƒ = 0.043 for type of load;F_3,33_ = 0.38, p = 0.766, ƒ = 0.187 for interaction of vision availability and type of load).

### Box Tilt during the Lifts

**Fig. 7:**
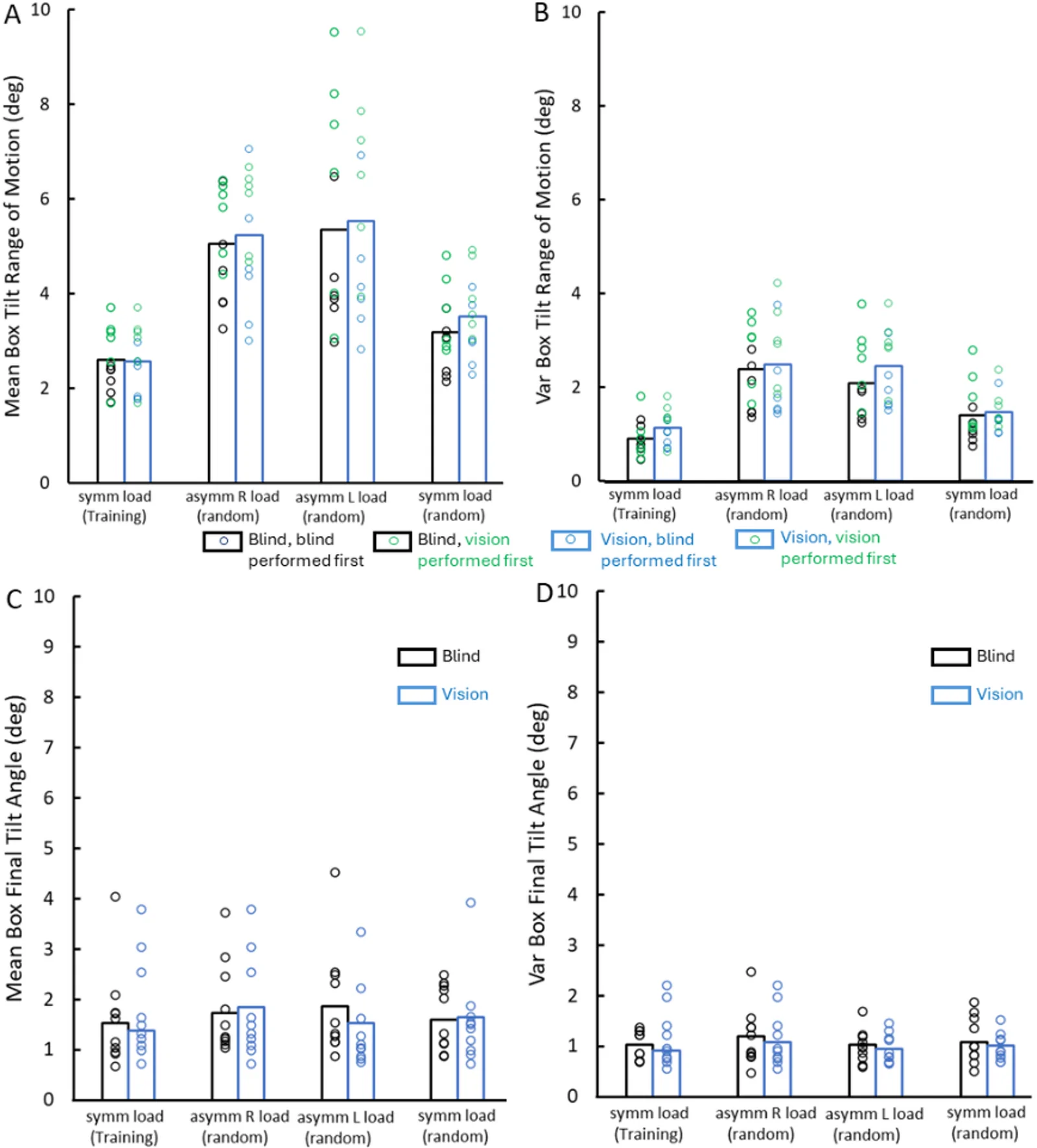
Mean box tilt range of motion (**A**), mean variability (S.D.) of box tilt range of motion (**B**) during the lifts, mean final tilt angle (**C**) and mean variability (S.D.) of final tilt angles, Each bar in A represents the mean across subjects of mean box tilt angle range of motion, The symbols represent individual subject means. Similarly, in B,C,D each bar represents the mean across subjects of the dependent variable and the symbols represent individual subject means. In A and B subjects who performed blindfolded first under no-vision conditions are the black circles (blind condition) and blue circles (vision condition) and subjects who performed with vision first are the green circles in both blind and vision conditions. Individual subject data are shown by the symbols plotted with each bar

As expected, the box tilted less throughout the lift when the load was distributed symmetrically than when it was distributed asymmetrically. This is evident in the examples from one subject (Fig. 2 – compare tilt in symmetrical and asymmetrical load lifts) and in the mean data on tilt range of motion (ROM) for all subjects (Fig. 7A). Notably, the range of box tilt motion averaged only 2.5°-3.5° when the box was symmetrically loaded and only 5°-5.5° when asymmetrically loaded (Fig. 7A). Similarly, trial-to-trial variability of box tilt ROM was clearly lower when lifting the box while symmetrically loaded (Fig. 7B). Performing without vision first was associated with slightly lower tilt ROM (Fig. 7A,F_1,10_ = 5.61, p = 0.039, ƒ = 0.749) and slightly lower variability of tilt ROM (Fig. 7B, F_1,10_ = 7.1, p = 0.018, ƒ = 0.893) in both vision conditions. As expected, both tilt ROM and variability of tilt ROM were lowest with symmetrical loads during training, slightly higher with symmetrical loads in random conditions and clearly higher with asymmetrical loads (Fig. 7, tilt ROM: F_3,30_ = 33.38, p < 0.001, ƒ = 1.275;p < 0.05 on post-hoc tests; variability of tilt ROM: F_3,30_ = 39.98, p < 0.001,, ƒ = 1.366; p < 0.05 on post-hoc tests). Tilt ROM and variability of tilt ROM were similar whether vision was available or not (tilt ROM: F_1,10_ = 1.09, p < 0.321,, ƒ = 0.330; S.D. of tilt ROM: F_1,10_ = 3.23,p = 0.103, ƒ = 0.568). There were no interaction effects involving vision availability on tilt ROM or variability of tilt ROM (p > 0.226), but there was an order x type of load interaction effect on tilt ROM (F_3,30_ = 4.11, p = 0.022, ƒ = 0.576).

Box tilt averaged less than 2° at movement termination in all lifting conditions (Fig. 7C) and was similar when the box was symmetrically and asymmetrically loaded (Fig. 7C, F_3,33_ = 1.96, p = 0.145, ƒ = 0.422) and was also similar with and without vision (Fig.7C, F_1,11_ = 0.35, p = 0.566, ƒ = 0.179). Variability of box tilt at movement end averaged less than 1.1° and was similar in the different loading conditions (Fig. 7D, F_3,33_ = 0.47, p = 0.443, ƒ = 0.281) and with and without vision (Fig. 7D, F_1,11_ = 2.79, p = 0.123, ƒ = 0.504). There were no vision x load interaction effects for box tilt or variability of box tilt at movement termination (p > 0.169).

### Load asymmetry transitions

**Fig. 8:**
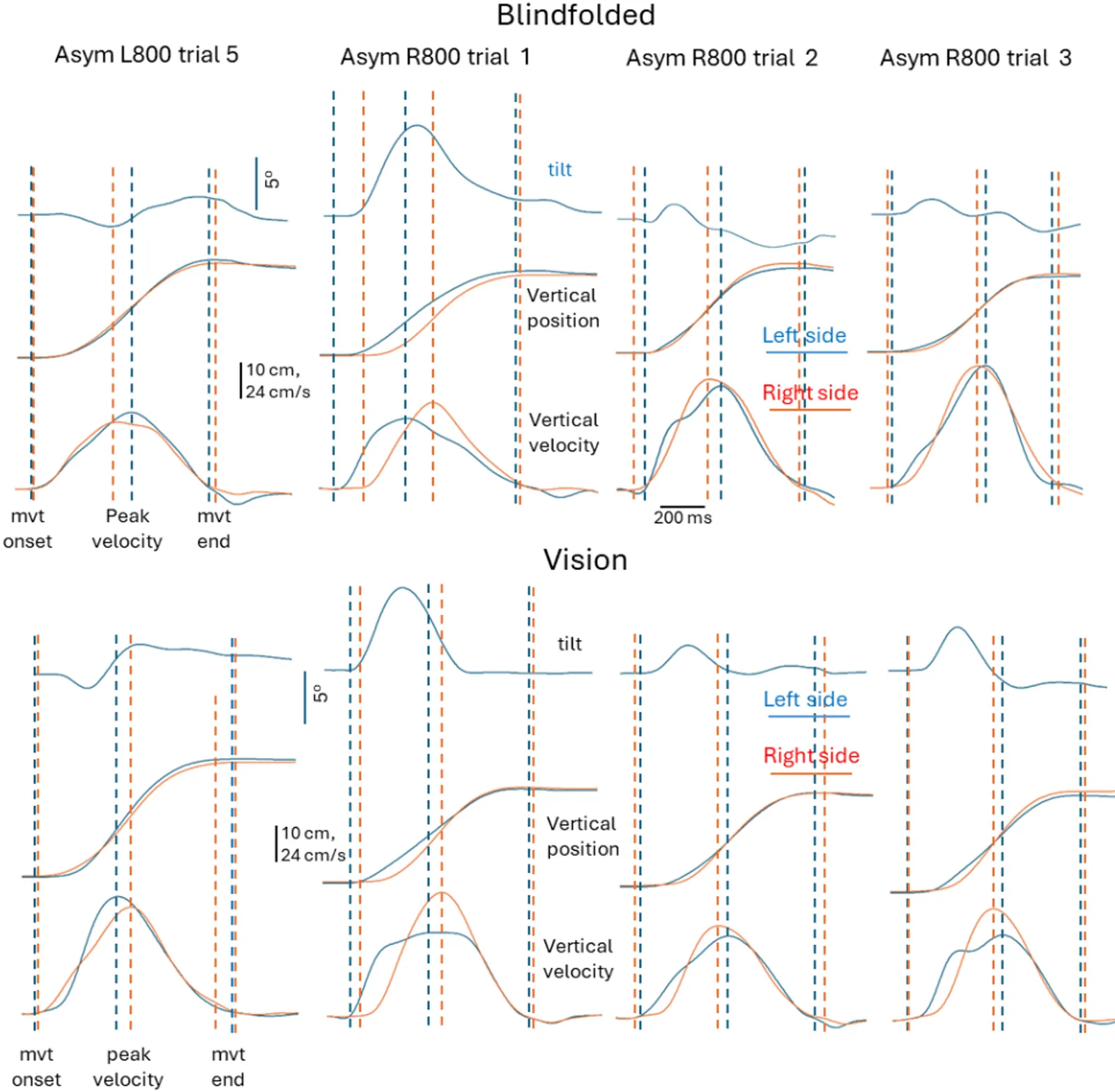
Examples of vertical position and velocity profiles of the right and left sides of the box and tilt angle of the box during lifts under transitions of load symmetry from 800 g on the left side of the box to 800 g on the right side of the box in two subjects. On the left is the final trial of 5 consecutive lifts with 800 g on the left side of the box. Note the well-synchronized lifts of the left and right sides of the box with minimal box tilt. The first three lifts after the 800 go load was moved to the right side of the box are also shown. Note the less-synchronized lift of the left and right sides and large box tilt in the 1st trial with the new load asymmetry and the improved synchrony and lower tilts in the 2nd and 3rd lifts. Movement onsets, times of peak velocity and movement terminations are shown as dotted lines

**Fig. 9:**
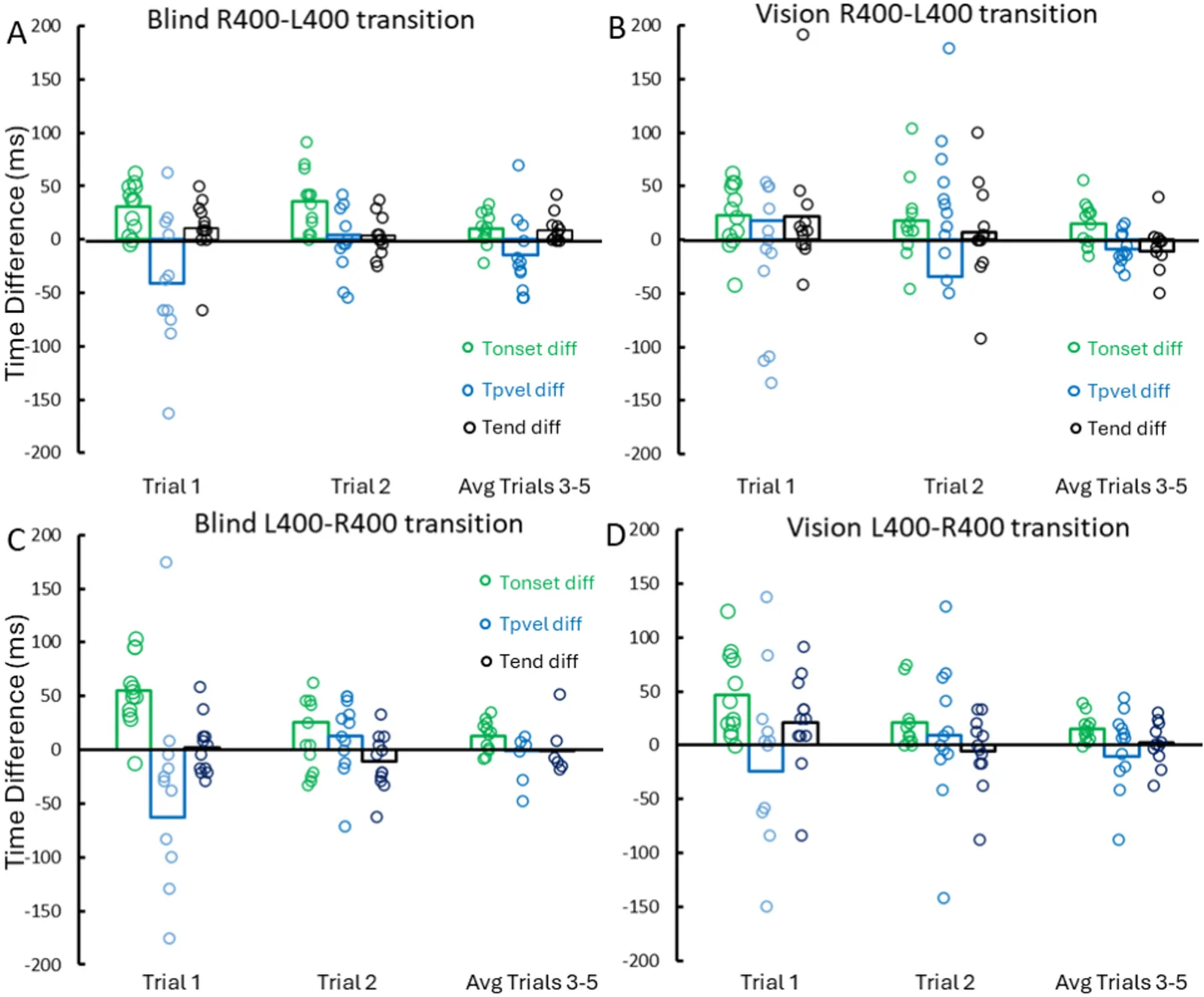
Temporal synchrony after load transitions from asymmetrical loads on one side of the box to 400 g asymmetrical loads on the other side of the box. Each bar is the mean difference in onset time, time of peak velocity or end time of movements in the first trial (green), 2nd trial (blue) or average of last 3 trials (black) after the transition. The plotted symbols are individual subject data. The title of each graph in A-D denotes the visual condition and type of transition. Abbreviations: R400-L400 = right asymmetrical 400 g load to left asymmetrical 400 g load, L400 – R400 = left asymmetrical 400 g load to left asymmetrical 400 g load

**Fig. 10:**
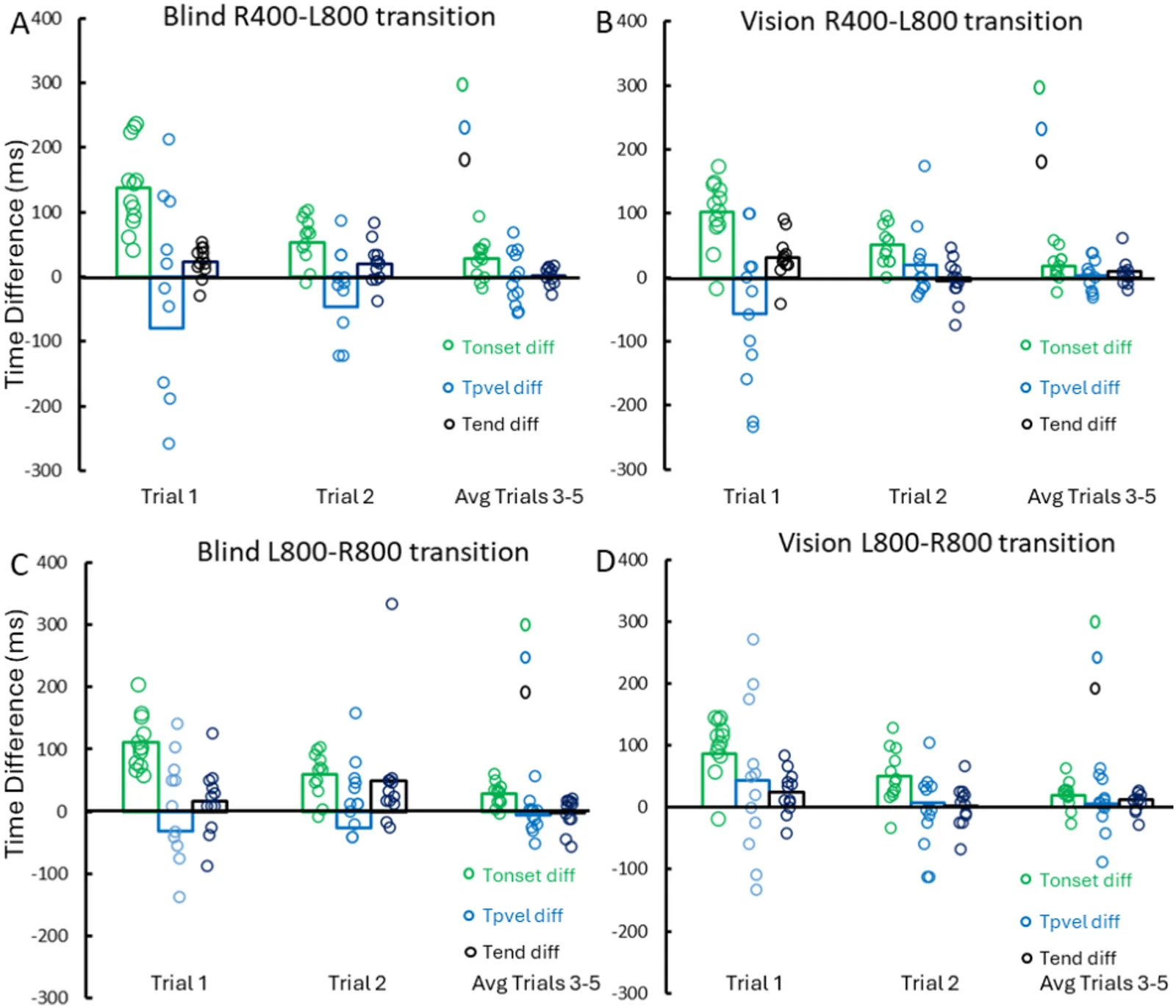
Temporal synchrony after load transitions from asymmetrical loads on one side of the box to 800 g asymmetrical loads on the other side of the box. Each bar is the mean difference in onset time, time of peak velocity or end time of movements in the first trial (green), 2nd trial (blue) or average of last 3 trials (black) after the transition. The plotted symbols are individual subject data. The title of each graph in A-D denotes the visual condition and type of transition. Abbreviations: R400-L800 = right asymmetrical 400 g load to left asymmetrical 800 g load, L800 – R800 = left asymmetrical 800 g load to left asymmetrical 800 g load

In general, transitions of load symmetry (i.e., heavier side of box switched from left to right or vice-versa) were associated with delays in movement onset and time of peak velocity of the heavier side, but movement terminations were well-synchronized (i.e., within about 50 ms), on the first lift. This is clearly shown in the examples from one subject (Fig. 8) and was also evident in all subjects (Figs. 9,10). On the second trial with the new asymmetrical load, the delay in movement onset for lifting the heavier side was usually decreased while peak velocity times became synchronized along with movement terminations (Figs. 9,10). Further decreases in delay of lift onset and peak velocity were observed in trials 3–5 such that the movements of two sides became approximately temporally synchronized (Figs. 8,9,10).

**Fig. 11:**
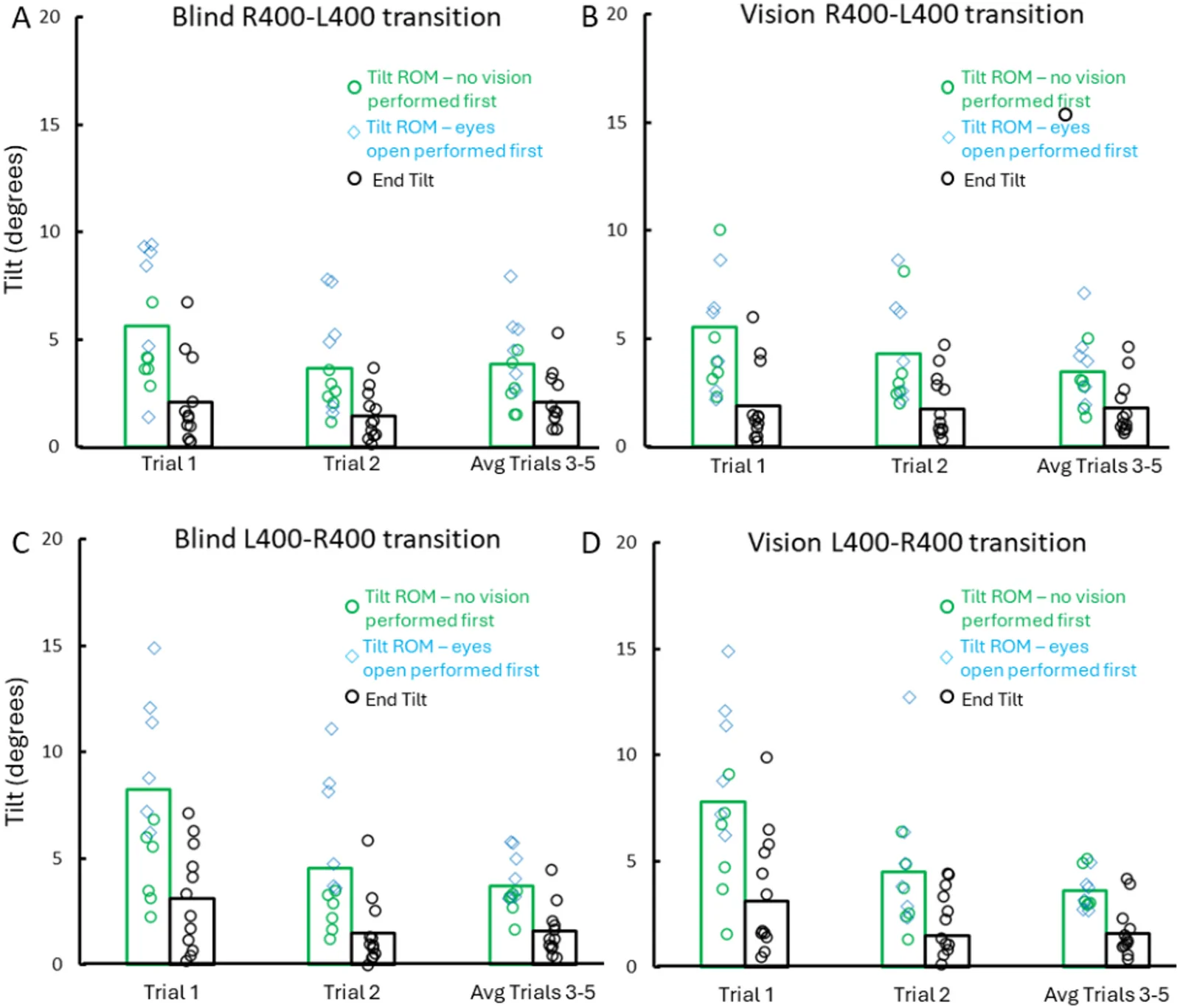
Mean box tilt range of motion and mean final box tilt angle in the 1st and 2nd trials and the average of trials 3–5 after transitions of 400 g load symmetry. Each bar represents the mean for 12 subjects of mean box tilt angle range of motion (green bars) or mean final box tilt angle (black bars). The plotted symbols are individual subject data. For tilt ROM, the blue diamonds are subject means when vision allowed was performed first and the green circles are subject means when vision not allowed was performed first. The title of each graph in A-D denotes the visual condition and type of transition. Abbreviations: R400-L400 = right asymmetrical 400 g load to left asymmetrical 400 g load, L400 – R400 = left asymmetrical 400 g load to left asymmetrical 400 g load

**Fig. 12:**
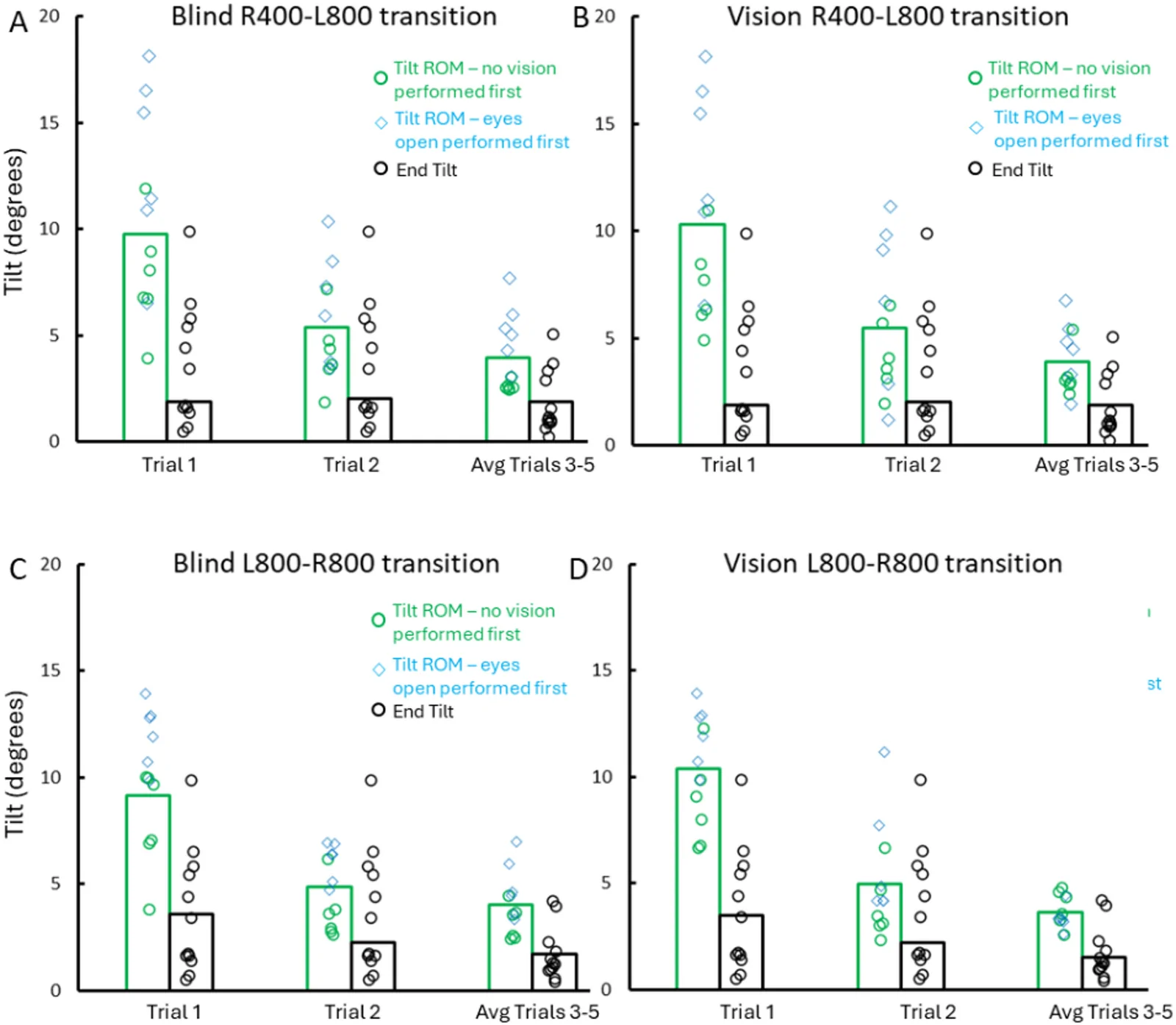
Mean box tilt range of motion and mean final box tilt angle in the 1st and 2nd trials and the average of trials 3–5 after transitions of 800 g load symmetry as indicated in the title for each graph. Each bar represents the mean across subjects of mean box tilt angle range of motion or mean final box tilt angle, The plotted symbols are individual subject data. For tilt ROM, the blue diamonds are subject means when vision allowed was performed first and the green circles are subject means when vision not allowed was performed first. The title of each graph in A-D denotes the visual condition and type of transition. Abbreviations: R400-L800 = right asymmetrical 400 g load to left asymmetrical 800 g load, L800 – R800 = left asymmetrical 800 g load to left asymmetrical 800 g load

The range of box tilt motion was usually relatively large on the first lift after a transition but was corrected during the latter part of the lift such that the box was nearly level at movement termination (e.g., Fig. 8). Data from all subjects confirmed these observations as tilt range of motion averaged 5–10° on the first lift after the transition but averaged only 1 – 3° at movement termination (Figs. 11,12). On subsequent lifts (trial 2, average of trials 3–5), the range of tilt motion decreased and there were small reductions in box tilt at movement termination. Statistical analyses confirming these general findings are presented below. In all but one dependent variable (tilt range of motion during the lift), there was no effect of order of performing with vision first versus blindfolded first as a main or interaction effect (p > 0.08). Thus, results of the three-way (transition type x vision x trial) repeated measures ANOVA are reported for most dependent variables.

After a transition to a new asymmetrical load the lift onset for the heavier side was usually delayed, whether vision was available or not, by 25 – 150 ms depending on the magnitude of the change in load symmetry. Specifically, changes in load symmetry from a 400 g load on one side to a 400 g load on the other side of the box resulted in 25–50 ms delays of lift onset for the heavier side (Fig. 7) whereas changes from 400 g or 800 g on one side to 800 g on the other side resulted in about 100 – 150 ms delays of lift onset for the heavier side (Fig. 8; F_3,33_ = 17.54, p < 0.001, ƒ = 1.263 for transition type main effect). However, there was considerable inter-subject variability such that some subjects performed almost synchronous lifts of the light and heavier sides especially when the load transition was to a 400g additional load on one side (Fig. 9). These onset delays usually decreased in the 2nd trial with the new load and, especially for transitions to 800 g loads on the other side, usually decreased further in trials 3–5 with the new load (Figs. 9,10). There was no effect of vision availability (F_1,11_ = 2.796, p = 0.123, ƒ = 0.523) and a significant transition type by trial interaction (F_6,66_ = 23.92, p < 0.001, ƒ = 1.475). Post-hoc comparisons confirmed that onset delays for lifting the heavier side of the box were larger when transitioning from a 400 g or 800 g load on one side to an 800 g load on the other side than when transitioning from a 400 g load on one side to a 400 g load on the other side (p < 0.05). The transition x trial interaction was due to larger reductions in onset times from trial 1 to trial 2 and to the average of trials 3–5 in transitions to 800 g loads on the other size than from a 400 g load on one side to a 400 g load on the other side.

Similar to the delay in movement onset for lifting the heavier side on the first trial after a transition in load symmetry, there was also a, usually smaller, delay in time of peak velocity (Figs. 9,10 F_3,33_ = 6.21, p = 0.002, ƒ = 0.752). However, this delay was mostly removed on the 2nd trial after the transition and remained near 0.0 ms on average in trials 3–5 (Figs. 9,10;). However, there was high inter-subject variability in peak velocity timing differences in the second trial in most transitions which was usually decreased in trials 3–5.

In contrast to movement onset and time of peak velocity, movement termination of the light and heavy sides was well-synchronized (within about 50 ms for most subjects) in the first lift following a transition of load symmetry (Figs. 8,9,10). Subsequent lifts were also well-synchronized but there was a trend for differences among transition types (F_3,33_ = 3.00,p = 0.073, ƒ = 0.522) and a transition type x vision interaction (F_3,33_, = 3.53, p = 0.025, ƒ = 0.523). However, there were no statistical differences in post-hoc tests of the interaction effect (p > 0.09) and it is clear that differences in heavy and light side movement termination were rather small for different transition types and vision conditions (Figs. 9,10).

Range of box tilt motion on the first lift after a transition of load symmetry averaged less than 10° for the transitions of load symmetry but were usually smaller when the load switched from 400 g on one side to the other side than when the load switched to 800 g on the other side (Figs. 11,12). Box tilt range of motion decreased to about 5° on average on the 2nd lift and averaged about 3° on lifts 3–5 with the new load symmetry. Statistically, there were main effects of order of performing first with vision vs. blindfolded (F_1,10_ = 9.59,p = 0.011, ƒ = 0.979), transition type (F_3,30_ = 8.57,p < 0.001, ƒ = 0.926) and trial (F_2,20_ = 130.58, p < 0.001, ƒ = 3.63), but no effect of vision (F_1,10_ = 0.09, p = 0.766, ƒ = 0.097). There were also two-way order x trial (F2,20 = 11.04,p < 0.001, ƒ = 1.051) and transition type x trial (F_6,60_ = 12.53, p < 0.001, ƒ = 1.119) interaction effects as well as an order x transition type x trial interaction effect (F_6,60_ = 3.18,p = 0.009, ƒ = 0.564), but no other interaction effects (p > 0.17). Post-hoc testing showed there were many statistical differences (i.e., p < 0.05) among the different conditions of performance order (vision first vs. blindfolded first), transition type and trial which were mainly due to the larger box tilt range of motion when subjects performed with vision first (Figs. 11,12) and the larger reductions in box tilt ROM from the first lift to the second lift when there was a transition to 800 g versus 400 g on the other side (Figs. 11,12).

Box tilt at the end of the lift averaged less than 4° in the first lift after a transition and tended to decrease on subsequent lifts (Fig. 11,12, F2,22 = 4.02, p = 0.063, ƒ = 0.604). Statistically, there was a main effect of transition type (F_3,33_ = 3.7, p = 0.032, ƒ = 0.58) and a transition type x trial x vision interaction (F_6,66_ = 2.82,p = 0.017, ƒ = 0.507). Post-hoc testing showed there were several differences (i.e., p < 0.05) among the different conditions of transition type, trial and vision but these differences averaged only 1–2°, showing that subjects generally performed very accurately in terms of leveling the box by the time of movement termination.

## Discussion

We observed that the kinematics of lifting a box with symmetrically and asymmetrically loaded weight distribution under conditions in which subjects did not know the weight or distribution of weight within the box were essentially the same with vision blocked and allowed. Moreover, similar performance was observed whether subjects first performed with vision or first performed without vision, except that box tilt ROM was slightly larger and more variable in subjects who performed with vision first. This was probably due to those subjects generally performing with larger tilt ROM as this was observed even in training lifts with symmetrical load distribution (Fig. 7A – see additional discussion below). Lifting a symmetrically loaded box was associated with a high degree of temporal synchrony of motion of the left and right sides with minimal tilt of the box during the lift both when vision was available and when it was blocked. This high temporal synchrony was expected based on previous findings of the grip and load forces for lifting two similar objects with the two hands, although actual lift kinematics were not reported (e.g., Serrien and Wiesendanger 2001; Steenbergen et al. 2008; Dimitriou and Buckingham 2018). In contrast, lifting an unexpected asymmetrically loaded box usually resulted in delayed lift onset of the heavier side and greater tilt of the box during the lift that were gradually corrected such that at movement termination the lighter and heavier sides motions ended nearly synchronously and the box was nearly level regardless of whether vision was blocked or not. This delayed onset of lifting the heavier side and lower synchrony of right and left side motions was also expected from previous findings of bimanual lifts of two objects because the applied load force increased for a longer duration to lift the unexpectedly heavier object on the first attempt, which should result in a later liftoff (Serrien and Wiesendanger 2001). Transitions of asymmetrical load distribution from the left to right side and vice-versa were associated with delayed onset for lifting the heavier side and large box tilt on the first attempt but termination of left and right side motions were nearly synchronous and the box was nearly level at termination of the lift. Again, there was no evidence that performance was better with vision than without. In subsequent attempts synchrony of the left and right side motions improved and box tilt ROM during the lift decreased without and with vision. Below we discuss contributions of proprioceptive/cutaneous and visual sensory information contributing to control of bimanual lifting of objects when weight and weight distribution are unknown in advance of the lift. 

When lifting an object with two hands, it is likely that parallel motor commands to the lifting muscles in each arm initiate and coordinate the lifting motion based on visual estimates of the muscle forces needed to lift the object or previous experience with the object. Such a control mechanism has been described previously in the idea that the two upper limbs become organized in a single coordinative structure (e.g., Kelso et al. 1979b; Wiesendanger et al. 1996). The first set of nine training trials with symmetrical weight distribution gave subjects experience lifting the box with the three levels of additional weight both when blindfolded and when vision was allowed. This likely permitted subjects to develop a set of muscle commands about the muscle forces needed to lift the box with different amounts of added weight. In the subsequent sets of 54 trials both weight and weight distribution were varied randomly. Without advance knowledge of the upcoming load or load distribution, subjects would probably use a plausible initial motor plan to deal with the middle (400 g added) weight. Planning based on the middle weight would ensure that the lowest weight was not lifted too quickly and the higher weight load would simply be delayed in lift onset as the applied muscle force could be increased to initiate the lift when it did not begin as expected. Thus, when the load distribution was symmetrical a highly synchronized motion of the two sides may occur due to the parallel muscle commands to each arm. In contrast, when there was an unexpected distribution of the load to one side, a delay in movement onset of that side would occur that would be rapidly detected by proprioceptors such as muscle spindles and cutaneous afferents surrounding upper limb joints (Collins et al. 2005) and in digit tips of the grasping hands in both blindfolded and eyes-open conditions. Stimulation of proprioceptive/cutaneous afferents after detection of lack of heavier side movement can elicit reflexive contractions of lifting muscles at short latencies whereas visual detection elicits muscle activity at much longer latencies as discussed in the next paragraph. Alpha-gamma coactivation during voluntary movements would result in strong stimulation of muscle spindles on the heavier side due to lack of shortening of extrafusal muscle fibers in parallel with intrafusal fibers of the muscle spindles in the lifting muscles for that side (Vallbo 1974; Prochazka 1981)..... Depending on the type of grasp, cutaneous stretch sensors in the digit tips and/or in the palms would detect differences in applied forces and motion of the lighter and heavier sides of the object.

We observed average delays of about 50 ms to initiate lifting the heavier side of the box after onset of the lighter side lift motion, with some subjects having shorter average delays (20–30 ms) while others exhibited average delays as long as 60–80 ms (Fig. 4A). In the first trial after large transitions in asymmetrical loading average delays averaged 80 – 140 ms (Fig. 10A,C). These delays to begin lifting motion of the heavier side are consistent with spinal and supraspinal responses to activate shoulder/elbow muscles to alter lifting forces after detection of the asymmetrical load. Due to the rather large variations in asymmetrical loading in the random variation of loading conditions, one would expect delays to lift asymmetrical loads that were 200 g higher to one side to be much shorter than delays to lift loads that were 400 or 800 g higher to one side. In such conditions It is likely that subjects with shorter average delays used a plausible initial motor plan with higher lifting muscle activation closer to that necessary to initiate lift of the box with the maximum additional load (800 g) while those with longer delays probably used a plausible initial motor plan with lifting muscle activation sufficient to lift the box with 400 g additional load. Indeed, the threshold for a muscle reflexive response to increase lift force for the more heavily loaded side would likely be shorter and reflex gain would be higher during stronger muscle contractions (e.g., Golkar et al. 2015) due to higher motor neuron excitability and higher gamma/beta motor neuron activity. This would result in a shorter delay to initiate lift of the heavier side via reflexive actions in lifting muscles.. Of course, voluntary increases in muscle activity to lift the heavier side probably also contribute to ensure that the box is nearly level at lift termination. These hypotheses could be tested in future experiments by recording lifting muscle activity along with lift forces applied by the two hands to each side of an unexpected asymmetrically loaded object.

The observation that there was substantial compensatory adaptation in initial motor commands to improve performance on the 2nd lift after a transition in load asymmetry from left to right and vice-versa (i.e., better synchronization of right and left sides onsets and time of peak velocity, less tilt during the 2nd lift than during the 1st lift). Further compensation occurred during lifts 3–5 following the transition (Figs. 9, 10, 11, 12). Tilt angle of the box at lift termination was minimal at the first lift in all transitions, except the L800-R800 transition both with and without vision (Figs. 11, 12). Notably, allowing vision did not result in better performance in adapting to transitions in load symmetry. Thus, proprioceptive inputs alone are sufficient to adapt applied lifting forces for successful performance of bimanual lifting of asymmetrically loaded objects. Overall, these findings regarding the effects of load asymmetry in the previous lift before a transition are similar to those reported for applied grip forces in precision grip bimanual lifts of two separate objects in that change from a heavy to a light weight on one side resulted in a higher peak grip force on the first trial followed by a much lower grip force on subsequent trials (Serrien and Wiesendanger 2001). Kinematic data on lift onset, etc. were not presented but it is likely that the lighter weight object was lifted earlier and with earlier peak upward velocity than the heavier weight object lifted by the other hand as was discussed in that report. The current findings are also consistent with reports on unimanual and bimanual lifts after unexpected changes of friction of the gripped surfaces (e.g., Westling and Johansson 1984; Edin et al. 1992). Specifically, when an unexpected change from silk (low friction) surface to sandpaper (high friction) occurs the grip force and safety margin (difference between grip force employed to hold an object and minimal grip force to prevent slipping) was higher than when the preceding lift had sandpaper surfaces (Westling and Johansson 1984). Moreover, this result was specific to individual digits when lifting a single object with the index and thumb and changes in surface friction occurred on only one side. Similar effects were observed in bimanual lifts with the right and left index tips when the unexpected change from silk to sandpaper occurred on one side only (Burstedt et al. 1997). The present findings differ from the effects of an unexpected lower friction on one side of an object which requires altered grip force and safety margin on that side for a successful lift whereas unexpected weight asymmetry requires increased lift force to the heavier side.

The finding that performing lifts first with vision allowed resulted in greater tilt ROM during lifts than when performing first without vision was not expected. We originally thought that there might be lower tilt ROM when vision was allowed while performing first as vision would provide additional sensory feedback to control tilt and the movements averaged about 700 ms duration (Fig. 3), thereby allowing sufficient time to use vision to modify coordination of the two hands to minimize box tilt. Practice of the task with vision performed first would then likely transfer to better performance under blindfolded conditions. However, this higher tilt ROM during the lift by subjects who performed with vision first was also observed under subsequent blindfolded conditions in the same subjects both in the random sequence of lifts and in the lifts following a transition of load asymmetry (Figs. 11, 12). We thus think that the subjects performing with vision first were simply less able to prevent excessive object tilt in all conditions than subjects who performed first without vision.

Although visual processing of object motion is clearly slower than proprioceptive processing of hand motion, the finding that allowing vision did not result in better performance in terms of synchronizing time of peak velocity and movement termination of the two sides or in reducing box tilt during the movement further demonstrates the effectiveness of proprioception in control of movement. Variability of these measures was also similar when performing blindfolded and with vision allowed. These findings are consistent with previous reports showing that proprioception provides similar or, in some cases, better accuracy compared to when vision is allowed during control of voluntary pointing movements of the right hand of right-handed individuals to stationary and moving body targets on the face and left hand (Capaday et al. 2013; Yoss et al. 2022; Darling and Yem 2023; Darling et al. 2024). Many previous reports of relatively poor accuracy in proprioceptive tasks have focused on perceptual tasks such as matching joint or segment angles of the two upper limbs, placing a body segment into horizontal and vertical orientations or reproducing a joint or body segment orientation or motion (e.g., Darling and Miller 1995; Darling and Hondzinski 1999; Goble and Brown 2009; Fuentes and Bastian 2010). Such tasks rely on conscious processing of proprioceptive information, which can result in relatively large errors in limb positioning and motion. In contrast, performing motor tasks may rely primarily on unconscious processing of proprioceptive information by spinal, brainstem and cerebellar nuclei which can result in rapid alterations in motor commands and are associated with relatively small movement errors (for additional discussion of these issues see Capaday et al. 2013; Darling et al. 2018; Coffman et al. 2021).

We were surprised that box tilt range of motion and at movement termination was not lower when vision was allowed. Moreover, trial-to-trial variability of these measures was similar when performing blindfolded and with vision allowed. Given the relatively long duration of these lifts and high visual acuity for detection of tilt from horizontal (e.g., Orban et al. 1984) there was sufficient time for vision to influence box tilt at later stages of the movements (Carlton 1981) to result in a more level box orientation than under blindfolded conditions. These findings further emphasize the precision of movement control provided by proprioceptive/cutaneous sensory inputs. Clearly, awareness of box tilt due to differences in height of the two hands from proprioceptive/cutaneous inputs as derived from joint angles of the two arms is sufficiently accurate to minimize box tilt during the movement and at movement termination. Indeed, both with and without vision box tilt ROM averaged only 5–6° with low variability (Fig. 7A,C) and the box was very close to level at movement termination (i.e., average box tilt of only 1–2° at movement termination with very low variability—Fig. 7B,D). Moreover, in the first trial after a very large transition in load symmetry (i.e., 400 or 800 g on one side to 800 g on the other side) box tilt ROM averaged only 10° and tilt at movement termination averaged less than 3° under both vision and blindfolded conditions (Fig. 12). This is remarkable performance of the task. Furthermore, the 2nd trial after a large transition of load symmetry resulted in a substantial reduction of box tilt ROM and, for the largest transition (800 g on one side to 800 g on the other side) even lower tilt at movement termination. Again, these performances were similar with and without vision available, further emphasizing the importance of proprioceptive/cutaneous inputs in the ability to adapt to the altered load asymmetry.

The primary limitations of the current study were lack of measurements of applied grip and lift forces and of EMG activities of upper limb muscles contributing to these forces. Thus, inferences related to neural control of these muscle activities and applied forces are indirect and limited to their effects on recorded motion of the two sides of the box. Lack of experimental conditions that remove or perturb proprioceptive and/or cutaneous sensations of hand motions and of applied forces also limit interpretations about the exact role of non-visual sensations in detecting motion and applied forces and using that information to control bimanual lifting. Another limitation is a potential confound of practice effects across the two vision conditions masking subtle vision effects. One might expect better performance in the condition performed second due to additional experience with the task. There was no evidence of practice effects in terms of temporal synchrony of left and right side lifts and box tilt at movement termination as there were no statistical effects of order of conditions for those variables. However, there was evidence of an order effect on tilt ROM and variability of tilt ROM during the lift as described in the Results section. Related to potential practice effects, there were slight reductions in tilt ROM and its variability (Fig. 7A,B – average reductions were less than 0.4°) when subjects performed without vision after performing with vision. In contrast, there were slight average increases in tilt ROM and tilt ROM variability (Fig. 7A,B, average increases were less than 0.3°) when subjects performed with vision after performing without vision. These very small changes in tilt ROM and its variability due to additional experience with the task are of no practical significance in relation to box tilt during the lift.

In conclusion, the present results clearly show that neurologically intact young adults do not need vision to assist in lifting an object with varied weight and weight distribution to a level orientation. This task can be performed accurately and with relatively little box tilt during motion and minimal tilt at movement termination with only proprioceptive/cutaneous sensory inputs available. These observations emphasize the importance of non-visual sensory inputs in control of a task similar to what may occur daily when lifting objects that may have asymmetric weight distribution. These results also emphasize the importance of developing therapies to restore proprioceptive deficits after neurological injuries such as stroke and traumatic brain injury to reduce dependence on vision for control of movements. Relatedly, the focus of such therapies should be on improving proprioceptive contributions to movement control, rather than on perception of limb orientation and motion.

## Data Availability

Data supporting the findings of this study, including single subject data, are available within the figures of this manuscript. Single subject data are available from the corresponding author upon reasonable request.
